# Triple Achilles Tendon Injury With Distal Avulsion, Proximal Rupture and Longitudinal Split in a Parkour Athlete: Case Report

**DOI:** 10.1002/ccr3.73325

**Published:** 2026-08-07

**Authors:** Alberto Grassi, Tosca Cerasoli, Claudio Rossi, Fabrizio Aggio, Francesco Della Villa, Stefano Zaffagnini

**Affiliations:** ^1^ Clinica Ortopedica E Traumatologica II IRCCS Istituto Ortopedico Rizzoli Bologna BO Italy; ^2^ Dipartimento di Scienze Biomediche e Neuromotorie DIBINEM Università di Bologna Bologna Italy; ^3^ Education and Research Department Isokinetic Medical Group, FIFA Medical Centre of Excellence Bologna Italy

**Keywords:** Achilles tendon rupture, distal avulsion, longitudinal split, multilevel tendon injury, parkour athlete, surgical repair

## Abstract

In unusual lesions of the Achilles Tendon, clinicians should be aware to perform an effective surgical technique aimed to restore anatomy and function. Medical history of the patients is crucial for understanding the type of traumatic mechanism. Unusual traumatic mechanisms may also present an unconventional injury pattern to repair.

## 1 Introduction

Achilles tendon rupture (ATR) is a common musculoskeletal injury with a multifactorial etiology that involves both intrinsic and extrinsic factors [1]. Its incidence has increased over recent decades [2], particularly among physically active individuals [1, 3, 4]. Although it typically affects middle‐aged men [5], younger athletes participating in sports involving explosive eccentric loading and rapid changes of direction are also at risk [6]. Emerging disciplines characterized by high‐impact dynamic movements, such as Parkour, may further increase the risk of ATR [7].

ATR most frequently occurs in the midportion of the tendon, 2 to 6 cm proximal to the calcaneal insertion, an area known for poor vascularity [6]. Less commonly, ruptures may occur proximally at the myotendinous junction or distally at its calcaneal insertion, either as an avulsion fracture involving a bony piece from the calcaneus or as a continuous sleeve avulsion rupture without any bony fragment [8]. Considering the variability in presentation, careful examination of atypical injuries is crucial to avoid misdiagnosis and suboptimal treatment, which may result in poor clinical outcomes.

In this report we present a complex and rare case of triple acute Achilles tendon rupture in a 25‐year‐old healthy amateur Parkour athlete, along with its surgical treatment and rehabilitation protocol.

## 2 Case Description

**FIGURE 1:**
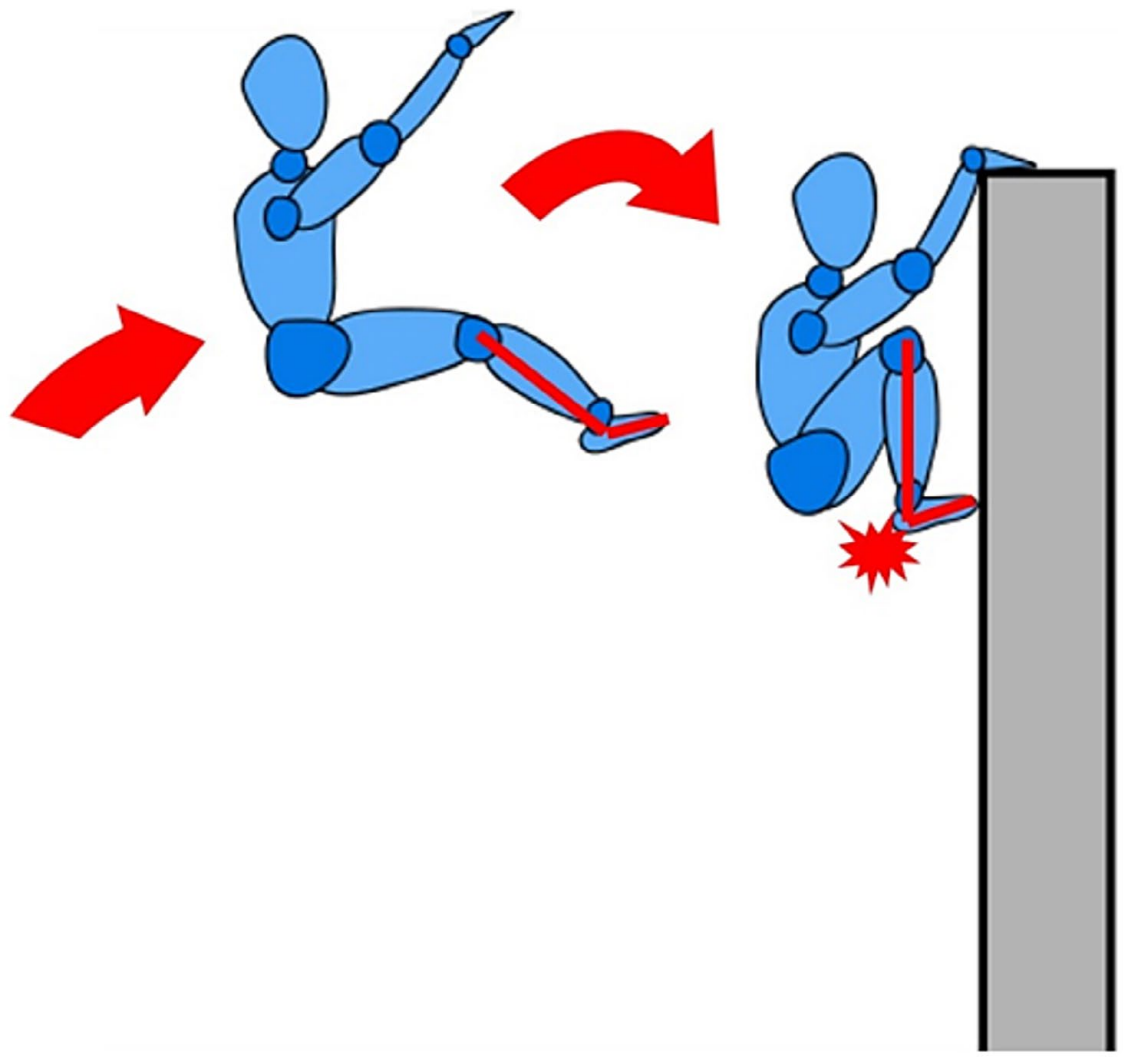
The “cat leap” in parkour involves jumping toward a vertical surface and catching it with the hands while the feet absorb impact against the wall. Upon contact, the hips and knees are deeply flexed, while the ankles are in forced dorsiflexion, placing significant eccentric load on the Achilles tendon. The rapid deceleration and high dorsiflexion angle increase tensile stress on the tendon, especially if joint control is insufficient.

A 25‐year‐old healthy amateur Parkour athlete was admitted to the Emergency Department of the Rizzoli Orthopedic Institute after sustaining an injury to his right ankle while performing Parkour. The patient reported a sudden forced ankle dorsiflexion while performing the “cat‐leap” trick (Figure 1), followed by extreme pain and inability to bear weight.

On clinical examination, the posterior aspect of the distal leg was swollen and painful during palpation. Lack of tension of the Achilles Tendon was noted; however no clear palpable gap was appreciated. The Thompson test was positive, consistent with an acute ATR. A foot radiograph was performed to rule out a calcaneal avulsion fracture. Given the unequivocal clinical diagnosis of ATR and the indication for urgent surgical treatment, no preoperative MRI was obtained, as it was not expected to alter the immediate management, and the patient was scheduled for open Achilles tendon repair on the same day.

### 2.1 Surgical Management

Surgery was performed by the senior surgeon (A.G.) using an open approach.

**FIGURE 2:**
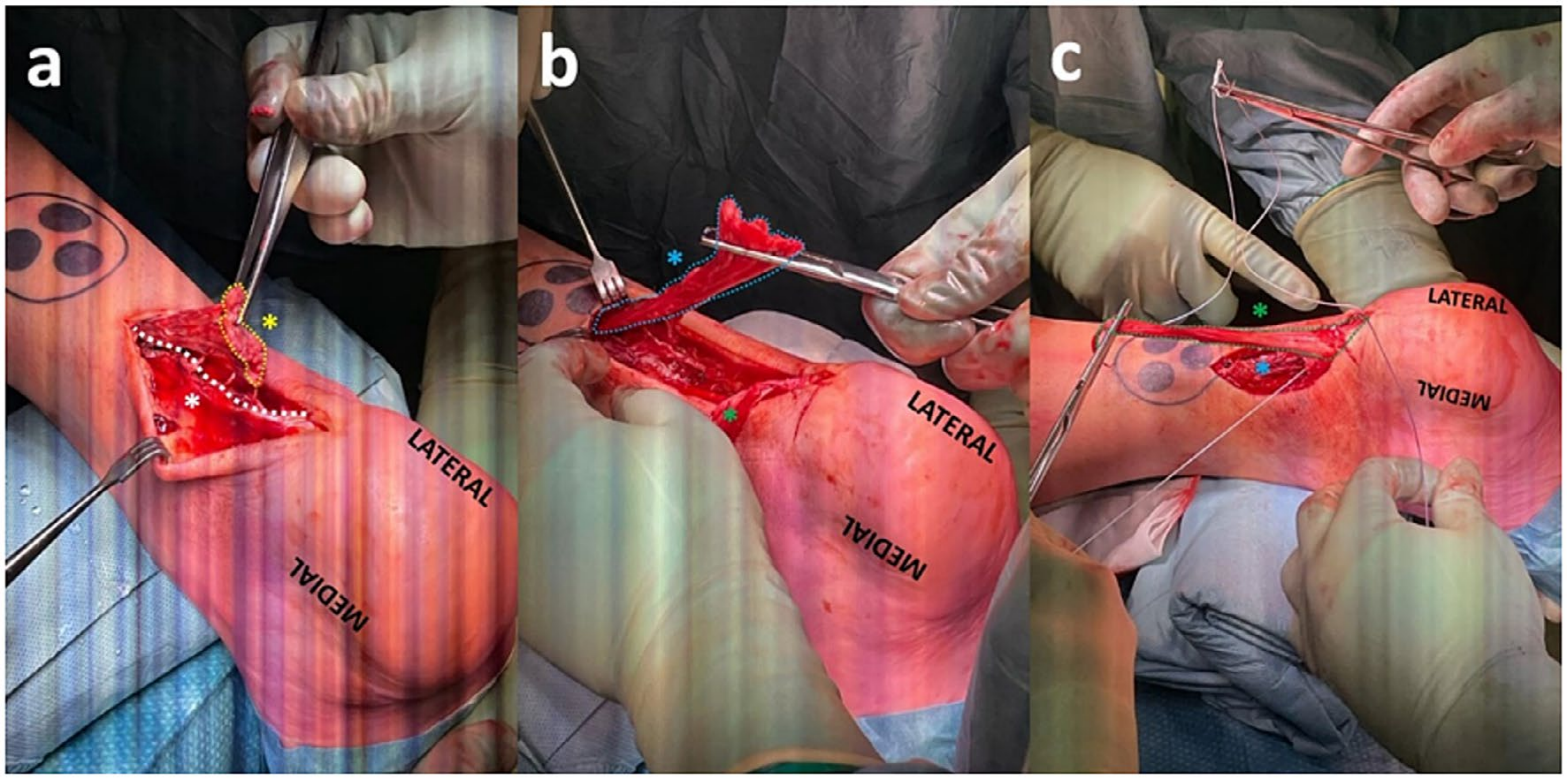
The proximal stump of the rupture (yellow asterisk) was identified and separated from the fascia (white asterisk) (a). When extending the incision to the calcaneal tuberosity a large part of the most superficial portion of the tendon was found to be still attached to the calcaneal tuberosity (green asterisk) and separated from the proximal and deep portion of the tendon (blue asterisk) (b). The most superficial fibers (green asterisk) had a length of nearly 15 cm and firm attachment to the calcaneus, separated from the deep fibers (blue asterisk) (c).

The patient was positioned prone under spinal anesthesia, and a tourniquet was applied. A longitudinal incision slightly medial to the Achilles tendon was performed in order to minimize the risk of injury to the sural nerve. After incising the swollen paratenon, a fresh hematoma was identified and evacuated. The proximal stump of the tendon was identified and debrided (Figure 2a). Based on its enlarged distal end, very distal location, and apparent absence of a distal stump, the lesion was initially interpreted as a calcaneal “sleeve” avulsion (Figure 2b).

However, when extending the incision to the calcaneal tuberosity to perform an osseous repair, a substantial portion of the superficial tendon fibers was found to remain attached to the calcaneal tuberosity. These fibers, which presented a width of nearly 3 cm, were followed proximally for about 15 cm and were noted to be detached at the myotendinous junction (Figure 2c).

A complex triple rupture was therefore diagnosed, consisting of: a distal calcaneal avulsion of the deep and lateral hemi‐tendon, a proximal rupture of the superficial and medial hemi‐tendon, and a longitudinal vertical split of the tendon.

**FIGURE 3:**
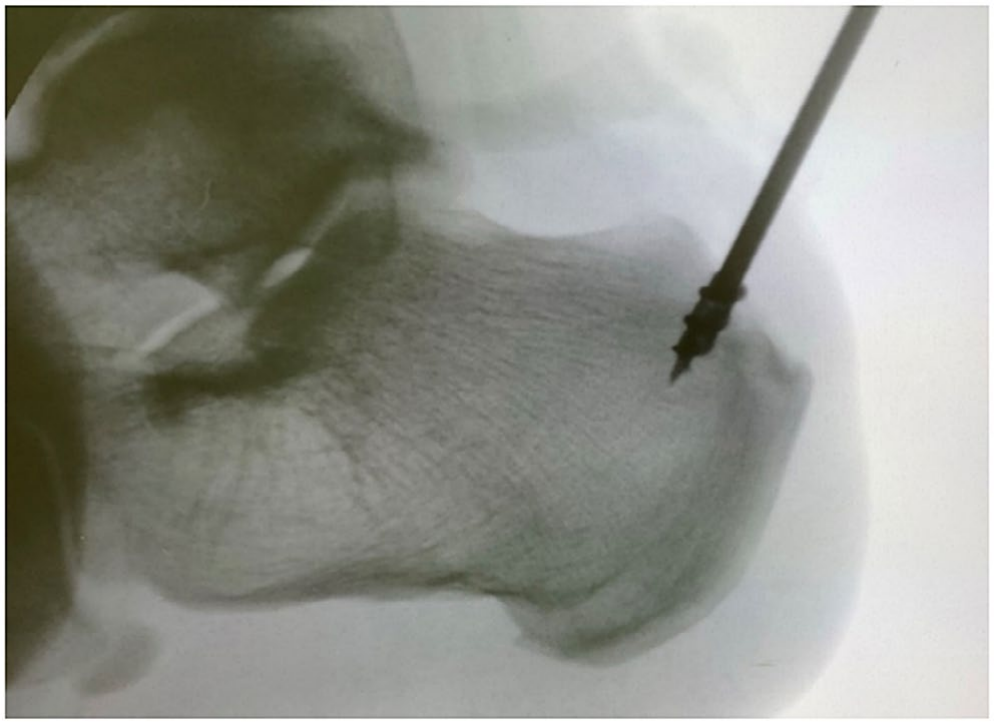
The deepest part of the tendon was re‐inserted to the calcaneal tuberosity with a metal anchor.

**FIGURE 4:**
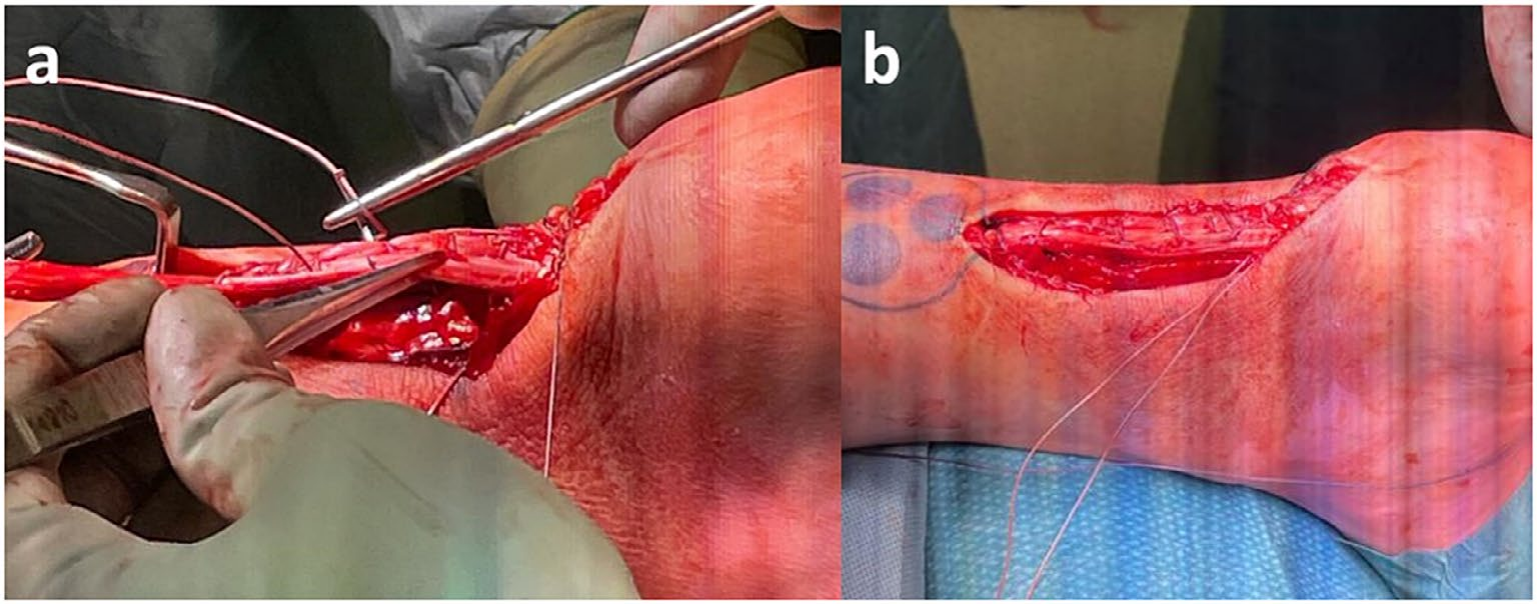
A side‐to‐side repair of the longitudinal split was performed with the non‐absorbable sutures from the anchor (a) restoring tendon continuity and tension (b).

Repair was performed using a combination of techniques. The deep portion of the tendon was whipstitched and re‐inserted to the calcaneal bone using a titanium 5.5 mm anchor (Figure 3). The medial portion was whipstitched with nonabsorbable N°2 sutures and repaired to the proximal aponeurosis. The longitudinal split was repaired with a continuous nonabsorbable suture integrated with the anchor fixation (Figure 4). The repair was performed with the foot in maximal plantarflexion to compensate for potential postoperative elongation. Skin was closed in layers, and a walking boot set at 20° plantarflexion was applied.

### 2.2 Post‐Operative Management

Wound care required careful monitoring due to the risk of delayed healing. Sutures were removed by the senior surgeon at 3 weeks postoperatively, followed by application of Steri‐Strips for an additional 5 days. Showering was permitted at 6 weeks postoperatively.

The rehabilitation protocol initially consisted of strict non‐weightbearing with continuous use of the walking boot (day and night) for 4 weeks (20° of plantar flexion). At 4 weeks, light touch weightbearing was allowed with the boot set at neutral. At 6 weeks, progressive partial to full weightbearing was permitted while maintaining the brace. From 6 weeks postoperatively, the patient was allowed to remove the brace for mobilization and strengthening exercises three times daily for 30 min.

At 6 weeks structured, criterion‐based rehabilitation program was initiated under physiotherapy supervision, progressing through defined phases each defined by specific clinical and functional milestones that had to be achieved before progressing to the next stage. At 8 weeks the brace was removed. Range of motion exercises were introduced under supervision, avoiding dorsiflexion beyond neutral and delaying stretching and eccentric loading until 12 weeks postoperatively to protect tendon healing. Around 14 weeks, the patient achieved a full body weight single‐leg heel raise.

At approximately 15 weeks, the patient achieved sufficient strength and control in the affected limb, and progressed to walking and then running on a treadmill at 6 km/h for at least 10 min without pain, swelling, or functional limitations. At around 21 weeks, the patient was able to perform at least 15 single‐leg heel raises on the operated limb, with a total count ≥ 80% compared to the contralateral side to move to the strength conditioning phase. In this phase, the patient performed running drills in a straight line without pain or swelling. Progression was allowed only if the following criteria were met: (1) adequate strength (single‐leg leg press ≥ 1.25× body weight or < 20% deficit compared to the contralateral side in isokinetic testing), and (2) quality execution of a single‐leg squat to 60° of knee flexion without compensatory movements.

Finally, the patient was reintroduced to field‐based running and neuromotor training. Complete return to sport was granted based on surgeon consensus, absence of pain or swelling during sport‐specific activities, and complete strength recovery (single‐leg leg press ≥ 1.5× body weight or 100% strength symmetry in isokinetic testing) and cardiovascular readiness. Throughout all phases, careful monitoring of inter‐limb compensatory strategies and functional symmetry was maintained to minimize the risk of re‐injury and ensure optimal performance outcomes (Table 1).

**TABLE 1:** Postoperative rehabilitation timeline and progression criteria.

Postop period	Brace/Weightbearing	Rehabilitation	Progression criteria
0–4 weeks	Non‐weightbearing. Walking boot locked at 20° plantarflexion (day and night)	Wound care and protection of the repair	Soft tissue healing
4–6 weeks	Touch weightbearing progressing to partial weightbearing. Boot at neutral position	Protected mobilization	No wound complications
6–8 weeks	Progressive full weightbearing with boot. Brace removed only for supervised exercises (30 min, three times/day)	Criterion‐based physiotherapy initiated. Gentle ROM avoiding dorsiflexion beyond neutral	Pain‐free progression
8–12 weeks	Brace discontinued	Progressive strengthening and ROM exercises. Stretching and eccentric loading delayed until week 12	Restoration of ankle mobility
15 weeks	Full weightbearing	Progressive strengthening. Progression from walking to treadmill running (6 km/h for ≥ 10 min)	Full body‐weight single‐leg heel raise achieved
21 weeks	Full activity progression	Strength and conditioning phase with straight‐line running drills	≥ 15 single‐leg heel raises and ≥ 80% limb symmetry. Leg press ≥ 1.25× body weight or < 20% isokinetic deficit
11 months	Return to unrestricted sport	Sport‐specific drills and neuromotor training	Surgeon clearance, absence of symptoms, full strength recovery (single‐leg leg press ≥ 1.5× body weight or 100% isokinetic symmetry)

### 2.3 Outcome

**FIGURE 5:**
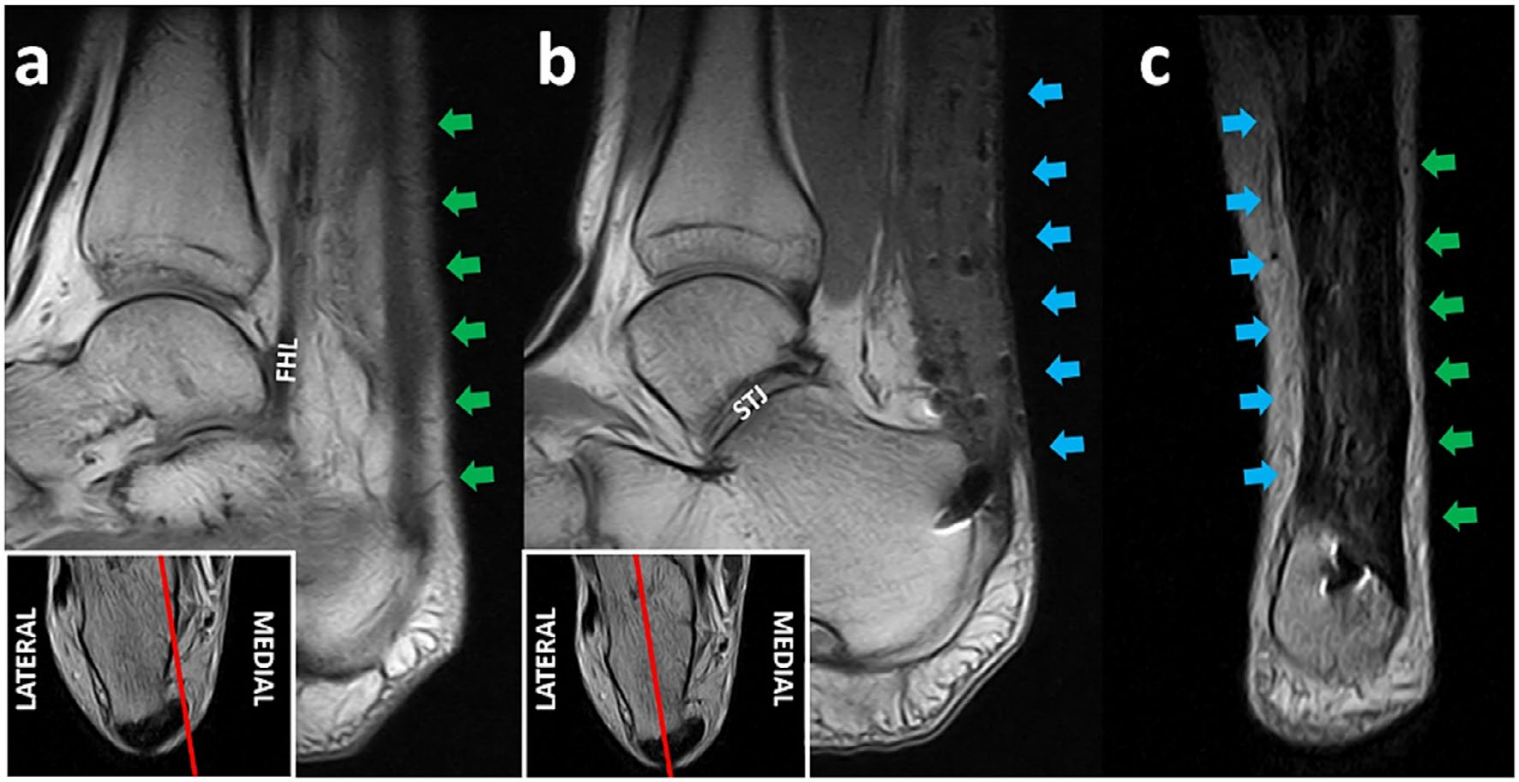
The 5 months MRI shows on sagittal plane the continuity of the most medial and superficial fibers (green arrows) with their intact native insertion (a) and the enlarged lateral and deep fibers (blue arrows) surgically reinserted to calcaneal tuberosity (b). Similar aspect is seen on the coronal plane (c).

**FIGURE 6:**
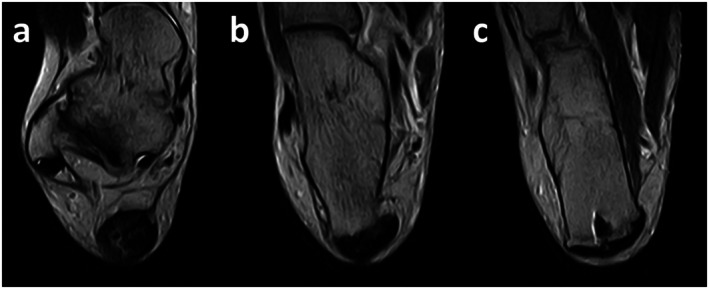
The 5 months MRI shows on axial plane the enlarged remodeled tendon in the most proximal slices (a), which becomes less thick close to the calcaneal insertion (b), especially at the level of more distal and superficial insertion (c).

No complications were reported during the postoperative period. An MRI performed 5 months after surgery demonstrated a continuous tendon; however with a thickened aspect and irregular signal intensity. The most medial and superficial part showed a regular ipointense signal and an anatomical insertion to the most distal and medial portion of the calcaneus (Figure 5a), while the lateral and deep portion had an irregular signal and thickness (Figure 5b,c). The most proximal and deep portion of the calcaneal insertion was thickened (Figure 6a), while the most distal portion and superficial respect to the skin was almost normal and well inserted into the calcaneal bone (Figure 6b,c).

The patient returned to parkour practice 11 months after injury. At a 3‐year follow‐up, the patient reported satisfaction with ankle function and had resumed full participation in sport without limitations.

## 3 Discussion

The present case report describes an unusual pattern of Achilles tendon rupture, highlighting the importance of recognizing atypical lesions in order to plan an appropriate surgical strategy aimed at restoring tendon anatomy and function.

A first‐key consideration relates to the mechanism of trauma. The young patient, a recreational parkour athlete, was able to clearly describe the dynamic of his injury during the “*cat le*ap” trick. This is a technique used in Parkour to land on a vertical object such as a ledge, a wall or a fence after a horizontal jump. During this task, the ankle sustains a forceful dorsiflexion at the moment of impact with the vertical surface; moreover, knee extension is required immediately afterward to climb the surface. The combination of high kinetic energy dissipation, forced dorsiflexion, and subsequent knee extension likely generated excessive tensile forces on the Achilles tendon, ultimately leading to rupture. This mechanism, characterized by passive stretching during a “deceleration” phase, differs from the more commonly reported mechanisms in sports such as soccer, where ruptures typically occur during active concentric contraction of the triceps surae during acceleration. These different biomechanics may partially explain the unconventional lesion pattern observed in this case.

**FIGURE 7:**
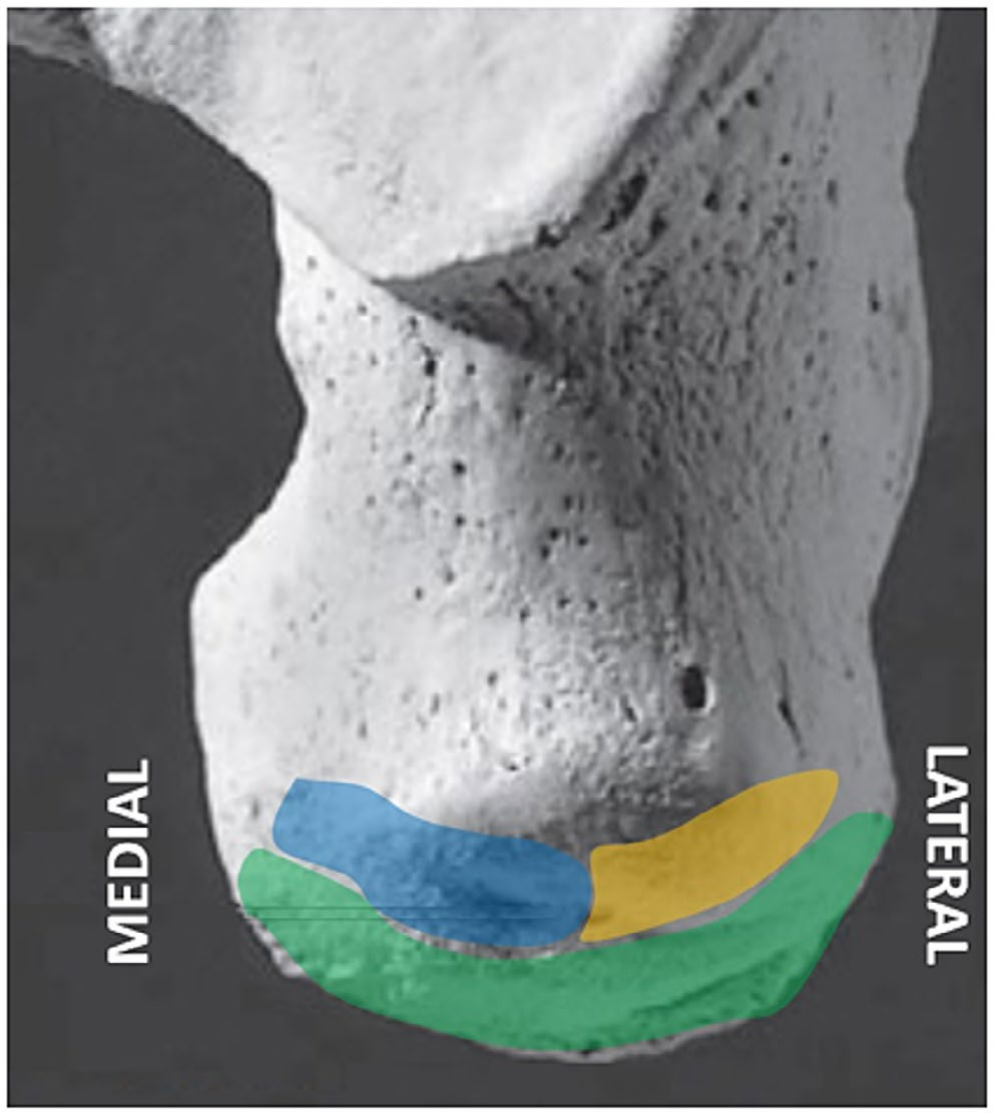
Schematic representation of the Achilles tendon calcaneal insertion, separating the fibers corresponding to the Medial Gastrocnemius (green area), Lateral Gastrocnemius (yellow area) and Soleus (blue area).

A second important consideration concerns tendon anatomy. Intraoperative findings revealed a clear distinction between the most superficial portion (in relation to the surgical view) of the tendon which remained attached to the calcaneus and the deep portion, which was avulsed. According to multiple anatomical studies, the fibers appearing superficial in the prone position correspond to the medial gastrocnemius insertion on the posterior calcaneus, whereas the deeper fibers correspond to the lateral gastrocnemius and soleus, inserting more anteriorly on the calcaneal tuberosity [3, 9, 10] (Figure 7). From an anatomical perspective, this case represents a selective avulsion of the lateral gastrocnemius and soleus portion of the Achilles Tendon, with a concurrent tear of the myotendinous junction of the medial gastrocnemius (which calcaneal insertion was almost perfectly intact) and associated a longitudinal split between these two portions.

This peculiar pattern suggests the presence of different “weak spots” within the musculotendinous complex: the myotendinous junction for the medial gastrocnemius, as suggested by the high incidence of muscle injuries in this area [11], and the calcaneal insertion for the soleus and lateral gastrocnemius. This configuration differs from the classical “sleeve avulsion” described in the literature. Bibbo et al. in 2003 [12] first reported this entity as a nonbony avulsion of the Achilles tendon from its calcaneal insertion, characterized by minimal residual tissue at the insertion site, often insufficient for direct repair. Similarly, Yang et al. [13] described complete detachment of the central portion of the tendon, with variable portion of medial and lateral tendon fibers remaining attached to the calcaneus.

To our knowledge, only one previous report has described a triple‐level Achilles tendon rupture. Saxena and Hofer reported a 57‐year‐old soccer referee presenting with a proximal rupture, a midsubstance rupture, and a distal avulsion associated with chronic insertional calcific tendinopathy [14]. In contrast, our patient was a young healthy athlete without pre‐existing tendon degeneration, and the injury occurred after a high‐energy Parkour maneuver. Moreover, the lesion configuration differed substantially, consisting of a selective distal avulsion of the deep and lateral tendon fibers, a proximal myotendinous rupture of the superficial and medial portion, and an associated longitudinal split. While the previously reported case likely reflected rupture through multiple degenerative weak points, the present case suggests that an unusual traumatic mechanism alone may generate a complex multilevel injury pattern in an otherwise healthy tendon. These differences expand the spectrum of triple‐level Achilles tendon injuries and emphasize the importance of individualized intraoperative assessment and repair.

The unusual injury configuration observed in this case also raises the question of preoperative imaging. Although MRI was not obtained preoperatively because of the urgent indication for surgery, advanced imaging might have been useful in retrospect to identify the unusual multilevel injury pattern. MRI remains the reference standard for characterizing tendon morphology and associated injuries, whereas high‐resolution ultrasound could also represent a valuable first‐line tool because it is inexpensive, readily available and capable of identifying discontinuity at multiple levels when performed by experienced operators [15]. Nevertheless, in the present case the intraoperative exploration ultimately provided the definitive diagnosis and guided the tailored repair.

From a surgical perspective, this case highlights the need for a tailored approach when managing atypical Achilles tendon injuries. The coexistence of distal avulsion, proximal rupture, and longitudinal splitting required a combined repair strategy addressing each component. The tendon avulsion could not have been repaired through the standard suture‐bridge approaches used for tendon reinsertion after calcaneoplasty because most of the calcaneal insertion was intact; therefore, a single anchor was used to reapproximate the avulsed part of the tendon and restore alignment and tension. This aided an optimal repair of the proximal lesion, which was followed by the final side‐to‐side repair of the longitudinal split.

Rehabilitation is a key component in the management of Achilles tendon rupture. Current evidence supports early functional rehabilitation with early weightbearing and ankle mobilization, typically starting at 2 weeks postoperatively, with dorsiflexion limited to neutral and eccentric loading delayed until approximately 12 weeks [16, 17, 18]. In this case, a more conservative approach was adopted due to lesion complexity, with non‐weightbearing for 4 weeks and full weightbearing at 6 weeks. Rehabilitation was conducted in a specialized setting using a criterion‐based progression, with advancement only after achieving specific strength and functional targets. Despite the initial delay, functional milestones and return to sport were achieved within timelines consistent with the literature [16, 17, 18]. These findings suggest that a protective early phase combined with criterion‐based rehabilitation may optimize outcomes in complex Achilles tendon injuries.

This case demonstrates that careful analysis of the injury mechanism and thorough intraoperative assessment are essential for identifying atypical rupture patterns. In cases of unusual Achilles tendon injuries, surgeons should maintain a high index of suspicion and be prepared to adapt the surgical technique to restore the specific anatomical and functional characteristics of the tendon.

The main limitations of this study are the lack of preoperative MRI, which could have provided additional information regarding the lesion pattern, and the absence of standardized patient‐reported outcome measures, which would have allowed a more objective assessment of the patient's functional recovery.

## 4 Conclusion

This case describes a rare and complex pattern of Achilles tendon rupture, combining distal avulsion, proximal rupture, and longitudinal splitting. Recognition of atypical injury mechanisms and lesion patterns is crucial to guide a tailored surgical strategy and optimize functional outcomes.

## Author Contributions

**Tosca Cerasoli:** writing – original draft, data curation, investigation, formal analysis. **Francesco Della Villa:** methodology, validation, supervision, writing – review and editing. **Stefano Zaffagnini:** supervision, data curation, conceptualization, methodology, validation. **Fabrizio Aggio:** validation, methodology, writing – review and editing. **Alberto Grassi:** conceptualization, investigation, methodology, validation, supervision, writing – original draft. **Claudio Rossi:** writing – original draft, writing – review and editing, investigation, formal analysis.

## Funding

The authors have nothing to report.

## Consent

Written informed consent was obtained from the patient for the use of their data in scientific research, in accordance with the Declaration of Helsinki.

## Conflicts of Interest

Alberto Grassi: DePuy and Smith and Nephew paid courses faculty member. Stefano Zaffagnini: DePuy, a Johnson & Johnson Company: Paid presenter or speaker; paid consultant. European Society of Sports Traumatology, Knee Surgery and Arthroscopy (ESSKA): Board or committee member. International Society of Arthroscopy, Knee Surgery, and Orthopedic Sports Medicine (ISAKOS): Board or committee member. Journal of Experimental Orthopedics (JEO): Editorial or governing board. Smith & Nephew: Paid presenter or speaker; paid consultant. All other authors declare no conflicts of interest related to this study.

## Data Availability

The data that support the findings of this study are available on request from the corresponding author.
